# Effects of Systemic Rehabilitation Nursing Combined with WeChat Publicity and Education on the Early Cognitive Function and Living Quality of the Patients with Cerebral Arterial Thrombosis

**DOI:** 10.1155/2022/7396950

**Published:** 2022-02-24

**Authors:** Zheng Jin, Fang Guo, Yan Li

**Affiliations:** Department of Neurosurgery, Tianjin Huanhu Hospital, Tianjin 300350, China

## Abstract

**Objective:**

To investigate the effects of systemic rehabilitation nursing combined with WeChat publicity and education on the early cognitive function and living quality of the patients with cerebral arterial thrombosis.

**Methods:**

Ninety-two patients with cerebral arterial thrombosis treated in our hospital (January 2019–January 2021) were selected as the research objects and equably divided into control group and study group according to their nursing model, with 46 patients in each group. The control group received routine nursing, while the study group received systemic rehabilitation nursing combined with WeChat publicity and education based on the routine nursing. The early cognitive functions, living quality, and other observation indexes of the two groups after nursing were compared, and the intervention effects were evaluated.

**Results:**

No statistical difference in the general data was observed between the two groups (*P* > 0.05). The NIHSS (National Institutes of Health Stroke Scale) scores, Fugl-Meyer scores, and MoCA (Montreal Cognitive Assessment) scores of the two groups after nursing were all better than those before nursing, and the NIHSS score, Fugl-Meyer score, and MoCA score of the study group after nursing were better than those of the control group (*P* < 0.05). Compared with the control group, the study group had a remarkably higher excellent and good rate of daily living ability after nursing (*P* < 0.05) and prominently higher SIS (Sensory Index Score) after nursing (*P* < 0.05). The SIS included the scores of emotion, strength, ADL (activities of daily living), memory and thinking, hand function, communication and participation as well as the total score.

**Conclusion:**

The systemic rehabilitation nursing combined with WeChat publicity and education can effectively reduce the degree of neurological deficit of the patients with cerebral arterial thrombosis, improve their early cognitive function and motor function, and increase their daily living ability and prognostic living quality.

## 1. Introduction

Cerebral arterial thrombosis, as known as cerebral infarction, is one of the most common cerebrovascular diseases and features high incidence, mutilation rate, and mortality. Its incidence accounts for about 70% of acute cerebrovascular diseases [1–3]. After the attack of cerebral arterial thrombosis, the patients are generally characterized by focal neurologic impairment, sensory disturbance, hemiplegia, aphasia, and ataxia, and the patients in severe condition may suffer from brain hernia or brain death. According to statistics, after the acute treatment stage, more than 75% of patients need to receive rehabilitation training to improve their living quality and limb movement function. The 6 months after the acute stage are the best rehabilitation period for the patients. If the patients are given professional guidance from the medical staff and professional and systemic rehabilitation nursing, they will probably restore the function of voluntary movement [4–7]. However, it is difficult for the patients to endure various physical disorders caused by severe condition. Besides, the patients may also suffer from such negative emotions as anxiety and depression, which are adverse to their recovery. At the same time, the patients and their families' awareness of the disease is also of great importance to their recovery. The traditional mode of publicity and education is mainly carrying out oral education for patients when discharged and asking for the patients' rehabilitation through telephone follow-up after discharged. The substantive effect of publicity and education in such mode is little [8]. With the development of science and technology, WeChat publicity and education breaks the limitations of time and space, improves the effectiveness of communication, and is conducive to the nursing service in clinic. In order to improve the clinical nursing and service quality for the patients with cerebral arterial thrombosis, our hospital has established a systemic rehabilitation nursing model combined with WeChat publicity and education, attempting to ensure the patients' rehabilitation effects. At present, there are few relevant studies, and this paper conducts a retrospective analysis of the clinical cases, summarized as follows.

## 2. Research Method

### 2.1. Cases Screening

The patients were screened according to the study objectives and those who met the following criteria were included. (1) The patients met the clinical diagnostic criteria for cerebral arterial thrombosis and were confirmed by electrocardiogram and imaging examination. (2) The patients had stable vital signs after the acute stage treatment. (3) The patients were attacked by cerebral arterial thrombosis for the first time. (4) The patients had no history of mental illness or family heredity history. (5) The patients received systemic rehabilitation nursing, and the professional nursing staff guided the patients to do the rehabilitation exercise. (6) The patients' families were informed of the study and signed the informed consent. The following patients were excluded. (1) The patients had serious complications or malignant tumors. (2) The patients' families could not use WeChat. (3) The patients were complicated with subarachnoid hemorrhage (a kind of severe disease of cerebrovascular rupture), silent lacunar infarction (without the self-conscious nervous system symptoms), and irreversible neurological impairment (severe neurological impairment without the possibility of nerve repair). (4) The patients' case information was incomplete. (5) The patients were complicated with other severe organic diseases. (6) The patients had severe hypertension, diabetes, hyperlipidemia, and other underlying diseases. Ninety-two patients with cerebral arterial thrombosis treated in our hospital (January 2019–January 2021) were selected as the research objects of the retrospective analysis.

### 2.2. Cases Grouping

The 92 patients were equably divided into the control group and study group according to the nursing model, with 46 patients in each group. The control group received routine nursing, while the study group received systemic rehabilitation nursing combined with WeChat publicity and education based on routine nursing. The study was approved by the hospital ethics committee.

### 2.3. Methods

Routine nursing: the patients were all given symptomatic treatment, like thrombolysis, controlling blood glucose and blood pressure, decreasing intracranial pressure, preventing infection and deep vein thrombosis, and other treatment measures. Besides, they received nursing services in accordance with the standard nursing methods in the neurology department, and the nursing measures mainly included observing the patients' consciousness, physical activity, pain nursing, sputum excretion, and preventing risk factors (such as fall, infection, and complications). The traditional mode of publicity and education was implemented according to the patients' needs, and their families were informed of relevant matters needing attention, health knowledge about cerebral arterial thrombosis, and guidance on limb activity, etc. After discharged, the patients received telephone follow-up at regular intervals and were informed of reexamination [9].

Systemic rehabilitation nursing combined with WeChat publicity and education. If the patients had stable life signs after the acute treatment stage, they would receive the rehabilitation training, including the limbs' postures and changes, joint motions of limbs, and functional training. (1) Limbs' postures and changes. The patients took the supine position. A pillow was put under the diseased pelvis to support the pelvis and prevent it from uplifting and retracting. Another pillow was placed under the knee to keep the knee in flexion. The quilt should not be placed beside the patients' feet. A soft pillow was put on the patients' chest. The patients stretched the elbow joint in the diseased side upward to 100° and put it on the soft pillow. If the patients failed to stretch the elbow joint upward to 100°, they should put it above the heart. The patients flexed the affected leg and extended it to the front, and a soft pillow was put under the knees to keep the hip joint in the internally rotated and flexed position. Meanwhile, the feet should not hang in the air. The patients extended the shoulder in the diseased side as forward as possible and maintained the angle between the shoulder and the body ≥90°. The patients extended each joint of the upper limb in the diseased side, with a soft pillow beneath the back to prevent the body from tilting. The lower limb in the diseased side was in a striding position, with the healthy leg in front and the diseased leg in the back, and the healthy leg could be supported by a soft pillow. The patients changed the posture once every 2 hours in the daytime and once every 3 hours at night with the help of other people. (2) Joint motions of limbs. The joint motions of limbs included the adduction and abduction of the shoulder joints of the upper limbs, the extension and flexion movement of the elbow joints, wrist joint, finger joints and knee joints, shrugging movement of the unilateral shoulder, Bobath handclasp, and the abduction, flexion, extorsion, intortion, and extension of the hip joints, 2 times/day and 10–20 min/time. Such motions gradually transited to the active movement according to the patients' recovery [10]. (3) Functional training. The patients took the training of activities of daily living, including dressing themselves, feeding themselves, and relieving themselves. In the later period, the training gradually transited to standing balance exercise and diversionary and sit training as well as the stair climbing exercise as appropriate [11]. At the same time, the nursing intervention was conducted with WeChat. (1) The nursing team was established, and the WeChat public platform and communication group were created. The patients and their families were guided to subscribe the WeChat public account and enter the group, and they were informed of the function and importance of the WeChat public account and communication group. (2) Editing the nursing contents in the WeChat platform. The nursing knowledge about the health behaviors, stress coping, and daily functional training for the patients with cerebral arterial thrombosis was collected and sorted out, and relevant contents were released after being reexamined. The patients' families were told to pay attention to the contents. The nursing contents were pushed into the WeChat public account every Monday, Wednesday, and Friday and were sent to the communication group in links. After receiving the information, the patients and their families took time to study the educational contents and could ask questions in the WeChat group. One nursing staff was responsible for recording the questions and analyzing the key points and difficulties in the nursing so as to formulate the targeted interventions. (3) Answering questions. After answering questions in the form of text, picture, audio, and video to eliminate the patients and their families' doubts, the patients with good recovery were encouraged to share their recent recovery experiences and daily health behaviors. The nursing team made an in-depth analysis of the patients' rehabilitation, clarified the shortcomings of the self-care and home nursing, and made suggestions for improvement so as to continuously rationalize the nursing plan.

### 2.4. Observation Indexes

The general data included the age, sex, complications (diabetes, hypertension, increased intracranial pressure, and brain edema), disease types (vertebral artery stenosis, cerebral arterial spasm, and thrombosis), educational level, and other information.

The NIHSS (National Institutes of Health Stroke Scale) [12] was adopted to assess the patients' degree of neurological deficit before and after nursing. The NIHSS contained the assessment of consciousness, speech, movement, sensation, coordinate motor, eye movement, and visual field. The NIHSS score was 0–42 points, and higher scores indicated severer degrees of neurological deficit. The Fugl-Meyer scale (the part of motor function assessment) was adopted to assess the patients' motor condition. Thirty-three items were included in the scale, with a maximum score of 2 for each item, and the score was proportional to the patients' motor function. The MoCA scale was a frequently used rapid evaluation tool for cognitive impairment and included such dimensions as attention, executive function, memory, language function, visual structure skills, abstract thinking, calculability, and orientation. The total score of the MoCA scale was 30 points, with ≥26 points considered as normal, 18–26 points as mild cognitive impairment, 10–17 points as moderate cognitive impairment, and <10 points as severe cognitive impairment. Each item was evaluated three times, and the average value was calculated.

Daily living ability: the patients' daily living ability was evaluated by Barthel indexes, including 10 daily activity items, such as bathing, going to toilet, dressing, continence, and eating. Each item was scored 0, 5, 10, or 15 points according to 4 evaluation criteria and the patients' self-care ability, with the total score of 100 points. Four ranks of the excellent, good, fair, and poor were graded according to the score, and the excellent and good rate was calculated.

Living ability: the SIS (Sensory Index Score) scale [13] was used to evaluate the impact of cerebral arterial thrombosis on the lives of patients, and it included the dimensions of emotion, strength, ADL (activities of daily living), memory and thinking, hand function, communication and participation as well as the total score. This scale included 59 items and was mainly used to assess the patients' body recovery. The score was proportionate to the patients' living quality.

### 2.5. Statistical Treatment

This study adopted the SPSS22.0 software to process all the experimental data and calculate the intergroup differences and used GraphPad Prism 7 (GraphPad Software, San Diego, USA) to draw graphs. The research data included count data and measurement data. The count data were tested by *χ*^2^ and expressed by *n* (%). The measurement data were tested by *t* and expressed by ($\bar{x}\pm s$). When *P* < 0.05, the differences were considered statistically significant.

## 3. Results

### 3.1. General Data

No notable difference in age, sex, diabetes, hypertension, increased intracranial pressure, brain edema, disease types, educational level, and other general data was observed between the two groups (*P* > 0.05, Table 1).

### 3.2. Neurological Function

**Figure 1:**
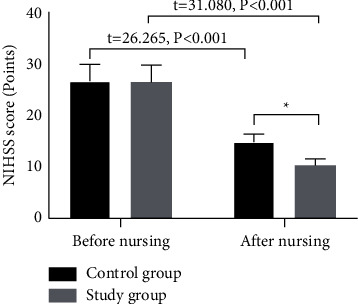
Analysis of the NIHSS score ($\bar{x}\pm s$). The abscissa indicated before nursing and after nursing, and the ordinate indicated the NIHSS score (points). The NIHSS scores in the control group before and after nursing were (26.71 ± 3.26) and (14.86 ± 1.55), respectively. The NIHSS scores in the study group before and after nursing were (26.55 ± 3.31) and (10.32 ± 1.26), respectively. ^*∗*^Presented the remarkable difference in the NIHSS score between the two groups after nursing (*t* = 15.415, *P* < 0.001).

The NIHSS scores in both groups showed decreasing tendency after nursing, and the study group had a lower NIHSS score than the control group after nursing (*P* < 0.05; Figure 1).

### 3.3. Motor Function

The Fugl-Meyer scores of the two groups increased after nursing, and the study group had a prominently higher Fugl-Meyer score after nursing than the control group (*P* < 0.05; Table 2).

### 3.4. Cognitive Function

**Figure 2:**
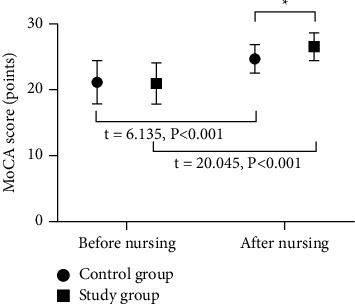
Analysis of the MoCA score ($\bar{x}\pm s$). The abscissa indicated before nursing and after nursing, and the ordinate indicated the MoCA score (points). The MoCA scores in the control group before and after nursing were (21.14 ± 3.27) and (24.68 ± 2.15), respectively. The MoCA scores in the study group before and after nursing were (20.96 ± 3.12) and (26.53 ± 2.10), respectively. ^*∗*^Presented the prominent difference in the MoCA score between the two groups after nursing (*t* = 4.175, *P* < 0.001).

The MoCA scores of the two groups increased after intervention, and the study group had a much higher MoCA score than the control group (*P* < 0.05; Figure 2).

### 3.5. Daily Living Ability

Compared with the control group, the study group had a remarkably higher excellent and good rate of daily living ability after nursing (*P* < 0.05). The intergroup difference had a statistical difference, as shown in Table 3.

### 3.6. Living Quality

Compared with the control group, the study group had a prominently higher SIS (in the dimensions of emotion, strength, ADL, memory and thinking, hand function, communication, and participation as well as the total score) after nursing (*P* < 0.05, Table 4).

## 4. Discussion

Because of the population aging in China, the patients with cerebral apoplexy are increasing day by day and getting younger. After the onset of cerebral arterial thrombosis, the patients' local cerebral tissue is in the state of hypoxia and ischemia, which then leads to hypoxia-ischemic lesions and neurologic impairment. According to statistics, about 70% of patients suffer from physical disability and cannot take care of themselves after the onset of cerebral arterial thrombosis, which seriously affects their living quality [14]. According to clinical investigations, the main reason for the high mutilation rate of elderly patients with cerebral arterial thrombosis is the neglect of rehabilitation nursing. In recent years, relevant studies have shown that the early rehabilitation nursing can effectively improve the patients' recovery, and the patients receiving complete systemic rehabilitation nursing have better recovery effects [5, 15]. In addition, based on secondary stroke prevention, our hospital expects to enhance the patients' health education by the WeChat publicity and education. Nursing staff should comprehensively analyze the causes of cerebral apoplexy, find the reversible causes that can be treated, and adjust risk factors that can be intervened so as to prevent cerebral apoplexy from happening again. The WeChat publicity and education can break the limitations of time and space of the secondary stroke prevention and nursing, improve the communication efficiency with patients, and extend the clinical publicity and education to the family so as to ensure that the patients can still receive effective intervention from the professional nursing team after discharged. As a result, the patients' daily living ability and prognostic quality of life can be improved. At present, there are some studies about the separate application effects of systemic rehabilitation nursing and WeChat publicity and education in patients with cerebral arterial thrombosis, but few studies investigate the combination of the two. Based on this, this paper analyzed the application situation of systemic rehabilitation nursing combined with WeChat publicity and education in 92 patients with cerebral arterial thrombosis treated in our hospital.

According to the study results, the NIHSS scores, Fugl-Meyer scores, and MoCA scores of the two groups after nursing were all better than those before nursing, and the NIHSS score, Fugl-Meyer score, and MoCA score of the study group after nursing were better than those of the control group (*P* < 0.05). Compared with the control group, the study group had a remarkably higher excellent and good rate of daily living ability after nursing (*P* < 0.05), which conformed to the report of Blackburm et al. [16]. Compared with the control group, the study group had prominently higher SIS (in the dimensions of emotion, strength, ADL, memory and thinking, hand function, communication, and participation as well as the total score) after nursing (*P* < 0.05), which further indicated that the systemic rehabilitation nursing combined with WeChat publicity and education could effectively improve the neurological deficit, limb movement function, and cognitive function of the patients with cerebral arterial thrombosis, further increasing their daily living ability and improving their living quality. The comprehensive analyses of the study are as follows. (1) The recovery of neurological function has great significance for the patients with cerebral apoplexy because the deterioration of neurological function directly impact their sensory, cognitive, and motor functions. In general, the damaged neural system can recover to a certain degree within a short period of time. Especially during the recovery of cerebral vessels, some nerve cells can regenerate or reorganize the structures and functions [17–20]. The optimal time period for rehabilitation is 6 months after the acute treatment stage, so the systematic rehabilitation nursing during this period can drive the nerve cells to produce new synapse and establish contact with the neuronal axon of collateral circulation. The reorganization of central neurons during this period can also promote the recovery of neurological functions. This study took the advantage of WeChat to enhance the intervention of clinical publicity and education, which was conducive to enhancing the patients and their families' awareness of the cerebral apoplexy and improving their cooperation with the systematic rehabilitation nursing. (2) The ultimate goal of patients' rehabilitation is to improve their living quality. Systematic rehabilitation nursing combined with WeChat publicity and education can help patients to establish a complete rehabilitation concept and improve their confidence. Besides, effective guidance on the limb exercise can prevent deep vein thrombosis and muscle atrophy and fundamentally improves the patients' limb movement function [21–24]. In this study, the patients transited from the limbs' postures and changes at first to active movement. The corresponding muscle groups were effectively stimulated during systematic rehabilitation nursing, which vitalized cell excitability and improved the patients' daily living abilities like swallowing, speech, dressing, and walking. As a result, patients' living quality was gradually improved. In conclusion, the systematic rehabilitation nursing combined with the WeChat publicity and education improves the cognitive function recovery and living quality of patients with cerebral arterial thrombosis, and its clinical practice effect deserves acknowledgement. The following points should be noted. (1) During the nursing intervention, whether the patients (the elderly patients in particular) have other complications should be noticed. If the patients frequently suffer from epilepsy, or have severe hydrocephalus, or their neurological function deteriorates, they will be considered unsuitable to receive the systematic rehabilitation nursing and be given the routine nursing dominated by treatment based on their condition. (2) The premise of WeChat publicity and education is the participation of the patients and their families. However, most of the patients with cerebral arterial thrombosis cannot participate in it in early stage of intervention. Therefore, it is important to ensure the effective participation of the patients' families in the publicity and education as well as the effectiveness of the output of relevant health knowledge in the patients' rehabilitation.

In conclusion, the systemic rehabilitation nursing combined with WeChat publicity and education can effectively reduce the degree of neurological deficit of the patients with cerebral arterial thrombosis, improve their early cognitive function and motor function, and increase their daily living ability and prognostic living quality.

## Figures and Tables

**Table 1:** Comparison of the general data (*n* = 46).

Observation index	Control group	Study group	*χ* ^2^/*t*	*P* value
Age (years)	53.84 ± 6.17	53.36 ± 6.20	0.372	0.711
Sex (male/female)	25/21	27/19	0.177	0.674
Complications				
Diabetes	9 (19.57)	7 (15.22)	0.303	0.582
Hypertension	28 (60.87)	29 (63.04)	0.046	0.830
Increased intracranial pressure	11 (23.91)	12 (20.09)	0.058	0.810
Brain edema	20 (43.48)	22 (47.83)	0.175	0.675
Barther score	60.11 ± 3.44	60.05 ± 3.37	0.085	0.933
Disease types				
Vertebral artery stenosis	9 (19.57)	8 (17.39)	0.072	0.788
Cerebral arterial spasm	12 (26.09)	14 (30.43)	0.215	0.643
Thrombosis	25 (54.35)	24 (52.17)	0.044	0.834
Educational level				
Under junior middle school	14 (30.43)	15 (32.61)	0.050	0.822
Middle school	22 (47.83)	24 (52.17)	0.174	0.677
Above senior high school	10 (21.74)	7 (15.22)	0.649	0.420

**Table 2:** Analysis of the Fugl-Meyer score ($\bar{x}\pm s$).

Group	*n*	Before nursing	After nursing
Control group	46	19.04 ± 4.79	25.26 ± 6.71
Study group	46	18.88 ± 5.01	36.54 ± 6.82
*t*		0.157	7.996
*P* value		0.876	<0.001

^∗^represented that the difference in the same group before and after nursing was of statistical significance (*P* < 0.05).

**Table 3:** Analysis of the daily living ability [*n* (%)].

Group	Excellent	Good	Fair	Poor	Excellent and good rate
Control group	4 (8.70)	13 (28.26)	19 (41.30)	10 (21.74)	17 (36.96)
Study group	10 (21.74)	22 (47.83)	10 (21.74)	4 (8.70)	32 (69.57)
*χ* ^2^					9.824
*P* value					0.002

**Table 4:** Analysis of SIS ($\bar{x}\pm s$).

Dimension	Control group	Study group	*t*	*P* value
Emotion	60.46 ± 3.91	64.72 ± 2.50	6.226	<0.001
Strength	44.13 ± 2.63	51.26 ± 3.25	11.567	<0.001
ADL	44.03 ± 2.57	49.08 ± 3.30	8.189	<0.001
Memory and thinking	72.50 ± 2.31	77.85 ± 2.24	11.277	<0.001
Hand function	32.85 ± 2.77	36.59 ± 2.84	6.394	<0.001
Communication	82.56 ± 3.42	87.05 ± 3.48	6.241	<0.001
Participation	24.55 ± 2.36	30.16 ± 2.74	10.522	<0.001
Total score	360.12 ± 24.71	397.24 ± 30.59	6.402	<0.001

## Data Availability

The data to support the findings of this study is available on reasonable request from the corresponding author.
